# Supplementary material for: Effectiveness and Safety of Linezolid Versus Vancomycin, Teicoplanin, or Daptomycin against Methicillin-Resistant Staphylococcus aureus Bacteremia: A Systematic Review and Meta-Analysis
Source: Antibiotics (Basel). 2023 Apr 2;12(4):697. doi: 10.3390/antibiotics12040697 (PMC10135165; doi:10.3390/antibiotics12040697)
Supplement: Supplementary file 1 [file antibiotics-12-00697-s001.zip › antibiotics-2271401-supplementary.pdf]

Supplementary Material

(A)

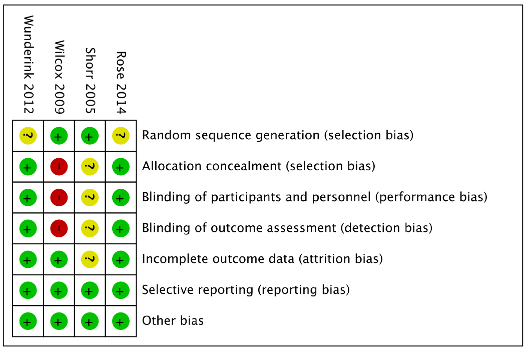

(B)

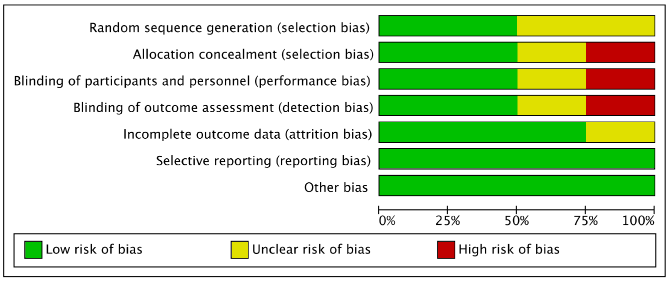

Figure S1. Risk of including RCT-based studies in the present meta-analysis. (A) Risk of bias summary: reviewers' judgments regarding each risk of bias domain for all included studies. (B) Risk of bias graph: reviewers' judgments regarding each risk of bias domain are presented as percentages across all included studies. ?: unclear risk of bias, –: high risk of bias, +: low risk of bias. RCT, randomized controlled trial.

(**A**)

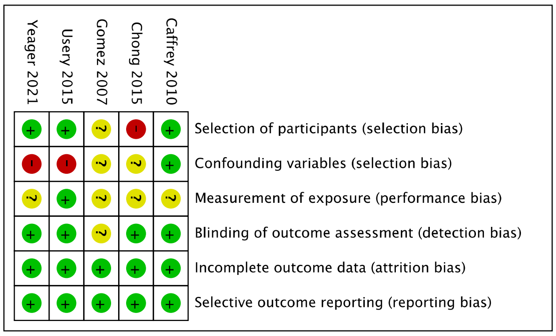

(**B**)

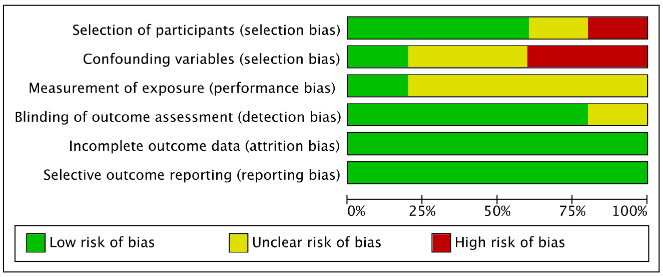

Figure S2. Risk of including CSs in the present meta-analysis. (A) Risk of bias summary: reviewers' judgments regarding each risk of bias domain for all the included studies. (B) Risk of bias graph: reviewers' judgments regarding each risk of bias domain are presented as percentages across all included studies. ?: unclear risk of bias, –: high risk of bias, +: low risk of bias. CSs, case-control and cohort studies.

Table S1. Terms used and results of searches in PubMed

No.	Search terms	Results
1	*Staphylococcus aureus*	144134
2	Methicillin-resistant *Staphylococcus aureus*	39034
3	Acute bacterial skin and skin structure infection	2900
4	Skin and skin structure infection	162175
5	Complicated skin and skin structure infection	43456
6	Skin and soft tissue infection	8711
7	Complicated skin and soft tissue infection	3004
8	Uncomplicated skin and soft tissue infection	183
9	Endocarditis	44446
10	Infective endocarditis	44443
11	Infectious endocarditis	10199
12	Bacterial endocarditis	27719
13	Bacteremia	52239
14	Bacteraemia	52239
15	Bacillaemia	25
16	Bacillemia	24
17	Bloodstream infection	214465
18	Linezolid	7572
19	Oxazolidinone	10607
20	Zyvox	7577
21	Daptomycin	3842
22	Lipopeptide	21894
23	Cubicin	3851
24	Vancomycin	35954
25	Teicoplanin	4585

26	Teichomycin	4585
27	Teicomycn	0
28	Targocid	4581
29	Glycopeptide	70049
30	(#1) OR (#2) OR (#3) OR (#4) OR (#5) OR (#6) OR (#7) OR (#8) OR (#9) OR (#10) OR (#11) OR (#12) OR (#13) OR (#14) OR (#15) OR (#16) OR (#17)	536868
31	(#18) OR (#19) OR (#20)	14245
32	(#21) OR (#22) OR (#23) OR (#24) OR (#25) OR (#26) OR (#27) OR (#28) OR (#29)	108866
33	(#30) AND (#31) AND (#32)	2708

Table S2. Terms used and results of searches in Web of Science.

No.	Search terms	Results
1	*Staphylococcus aureus*	160347
2	Methicillin-resistant *Staphylococcus aureus*	34798
3	Acute bacterial skin and skin structure infection	763
4	Skin and skin structure infection	5162
5	Complicated skin and skin structure infection	983
6	Skin and soft tissue infection	8370
7	Complicated skin and soft tissue infection	1142
8	uncomplicated skin and soft tissue infection	235
9	Endocarditis	36868
10	Infective endocarditis	15459
11	Infectious endocarditis	11506
12	Bacterial endocarditis	6629
13	Bacteremia	45127
14	Bacteraemia	45120
15	Bacillaemia	4
16	Bacillemia	25
17	Bloodstream infection	16204
18	Linezolid	7378
19	Oxazolidinone	3467
20	Zyvox	87
21	Daptomycin	4829
22	Lipopeptide	5065
23	Cubicin	69
24	Vancomycin	39711
25	Teicoplanin	4598

26	Teichomycin	41
27	Teicomycn	0
28	Targocid	8
29	Glycopeptide	9394
30	(#1) OR (#2) OR (#3) OR (#4) OR (#5) OR (#6) OR (#7) OR (#8) OR (#9) OR (#10) OR (#11) OR (#12) OR (#13) OR (#14) OR (#15) OR (#16) OR (#17)	242601
31	(#18) OR (#19) OR (#20)	9891
32	(#21) OR (#22) OR (#23) OR (#24) OR (#25) OR (#26) OR (#27) OR (#28) OR (#29)	53693
33	(#30) AND (#31) AND (#32)	2743

Table S3. Terms used and results of searches in the Cochrane Library.

No.	Search terms	Results
1	*Staphylococcus aureus*	4058
2	Methicillin-resistant *Staphylococcus aureus*	1364
3	Acute bacterial skin and skin structure infection	294
4	Skin and skin structure infection	848
5	Complicated skin and skin structure infection	195
6	Skin and soft tissue infection	944
7	Complicated skin and soft tissue infection	144
8	Uncomplicated skin and soft tissue infection	60
9	Endocarditis	807
10	Infective endocarditis	245
11	Infectious endocarditis	176
12	Bacterial endocarditis	416
13	Bacteremia	2932
14	Bacteraemia	2935
15	Bacillaemia	1
16	Bacillemia	1
17	Bloodstream infection	1372
18	Linezolid	616
19	Oxazolidinone	108
20	Zyvox	22
21	Daptomycin	224
22	Lipopeptide	56
23	Cubicin	19
24	Vancomycin	2402
25	Teicoplanin	355

26	Teichomycin	1
27	Teicomycn	0
28	Targocid	9
29	Glycopeptide	141
30	(#1) OR (#2) OR (#3) OR (#4) OR (#5) OR (#6) OR (#7) OR (#8) OR (#9) OR (#10) OR (#11) OR (#12) OR (#13) OR (#14) OR (#15) OR (#16) OR (#17)	9191
31	(#18) OR (#19) OR (#20)	652
32	(#21) OR (#22) OR (#23) OR (#24) OR (#25) OR (#26) OR (#27) OR (#28) OR (#29)	2758
33	(#30) AND (#31) AND (#32)	179

Table S4. Terms used and results of searches in ClinicalTrials.gov

No.	Search terms	Results
1	*Staphylococcus aureus*	820
2	Methicillin-resistant *Staphylococcus aureus*	342
3	Acute bacterial skin and skin structure infection	53
4	Skin and skin structure infection	1208
5	Complicated skin and skin structure infection	63
6	Skin and soft tissue infection	143
7	Complicated skin and soft tissue infection	22
8	Uncomplicated skin and soft tissue infection	10
9	Endocarditis	401
10	Infective endocarditis	139
11	Infectious endocarditis	120
12	Bacterial endocarditis	156
13	Bacteremia	849
14	Bacteraemia	849
15	Bacillaemia	1
16	Bacillemia	1
17	Bloodstream infection	5447
18	Linezolid	195
19	Oxazolidinone	52
20	Zyvox	195
21	Daptomycin	127
22	Lipopeptide	32
23	Cubicin	127
24	Vancomycin	685
25	Teicoplanin	48

26	Teichomycin	48
27	Teicomycn	0
28	Targocid	48
29	Glycopeptide	62
30	(#1) OR (#2) OR (#3) OR (#4) OR (#5) OR (#6) OR (#7) OR (#8) OR (#9) OR (#10) OR (#11) OR (#12) OR (#13) OR (#14) OR (#15) OR (#16) OR (#17)	7668
31	(#18) OR (#19) OR (#20)	233
32	(#21) OR (#22) OR (#23) OR (#24) OR (#25) OR (#26) OR (#27) OR (#28) OR (#29)	826
33	(#30) AND (#31) AND (#32)	42

Table S5. PRISMA 2020 Checklist

Section and Topic	Item #	Checklist item	Location where item is reported
**TITLE**			
Title	1	Identify the report as a systematic review.	Title
**ABSTRACT**			
Abstract	2	See the PRISMA 2020 for Abstracts checklist.	Abstract
**INTRODUCTION**			
Rationale	3	Describe the rationale for the review in the context of existing knowledge.	1–2
Objectives	4	Provide an explicit statement of the objective(s) or question(s) the review addresses.	2
**METHODS**			
Eligibility criteria	5	Specify the inclusion and exclusion criteria for the review and how studies were grouped for the syntheses.	2
Information sources	6	Specify all databases, registers, websites, organisations, reference lists and other sources searched or consulted to identify studies. Specify the date when each source was last searched or consulted.	2
Search strategy	7	Present the full search strategies for all databases, registers and websites, including any filters and limits used.	16; Tables S1–S4
Selection process	8	Specify the methods used to decide whether a study met the inclusion criteria of the review, including how many reviewers screened each record and each report retrieved, whether they worked independently, and if applicable, details of automation tools used in the process.	16
Data collection process	9	Specify the methods used to collect data from reports, including how many reviewers collected data from each report, whether they worked independently, any processes for obtaining or confirming data from study investigators, and if applicable, details of automation tools used in the process.	16
Data items	10a	List and define all outcomes for which data were sought. Specify whether all results that were compatible with each outcome domain in each study were sought (e.g. for all measures, time points, analyses), and if not, the methods used to decide which results to collect.	16
	10b	List and define all other variables for which data were sought (e.g. participant and intervention characteristics, funding sources). Describe any assumptions made about any missing or unclear information.	16
Study risk of bias assessment	11	Specify the methods used to assess risk of bias in the included studies, including details of the tool(s) used, how many reviewers assessed each study and whether they worked independently, and if applicable, details of automation tools used in the process.	16
Effect measures	12	Specify for each outcome the effect measure(s) (e.g. risk ratio, mean difference) used in the synthesis or presentation of results.	16–17
Synthesis methods	13a	Describe the processes used to decide which studies were eligible for each synthesis (e.g. tabulating the study intervention characteristics and comparing against the planned groups for each synthesis (item #5)).	16–17
	13b	Describe any methods required to prepare the data for presentation or synthesis, such as handling of missing summary statistics, or data conversions.	16–17
	13c	Describe any methods used to tabulate or visually display results of individual studies and syntheses.	16–17
	13d	Describe any methods used to synthesize results and provide a rationale for the choice(s). If meta-analysis was performed, describe the model(s), method(s) to identify the presence and extent of statistical heterogeneity, and software package(s) used.	16–17
	13e	Describe any methods used to explore possible causes of heterogeneity among study results (e.g. subgroup analysis, meta-regression).	Not applicable
	13f	Describe any sensitivity analyses conducted to assess robustness of the synthesized results.	Not applicable

Section and Topic	Item #	Checklist item	Location where item is reported
Reporting bias assessment	14	Describe any methods used to assess risk of bias due to missing results in a synthesis (arising from reporting biases).	Not applicable
Certainty assessment	15	Describe any methods used to assess certainty (or confidence) in the body of evidence for an outcome.	16
**RESULTS**			
Study selection	16a	Describe the results of the search and selection process, from the number of records identified in the search to the number of studies included in the review, ideally using a flow diagram.	3; Fig. 1
	16b	Cite studies that might appear to meet the inclusion criteria, but which were excluded, and explain why they were excluded.	Fig. 1
Study characteristics	17	Cite each included study and present its characteristics.	Table 1
Risk of bias in studies	18	Present assessments of risk of bias for each included study.	Figs. S1–S2
Results of individual studies	19	For all outcomes, present, for each study: (a) summary statistics for each group (where appropriate) and (b) an effect estimate and its precision (e.g. confidence/credible interval), ideally using structured tables or plots.	Figs. 2–6
Results of syntheses	20a	For each synthesis, briefly summarise the characteristics and risk of bias among contributing studies.	10–13
	20b	Present results of all statistical syntheses conducted. If meta-analysis was done, present for each the summary estimate and its precision (e.g. confidence/credible interval) and measures of statistical heterogeneity. If comparing groups, describe the direction of the effect.	10–13
	20c	Present results of all investigations of possible causes of heterogeneity among study results.	Not applicable
	20d	Present results of all sensitivity analyses conducted to assess the robustness of the synthesized results.	Not applicable
Reporting biases	21	Present assessments of risk of bias due to missing results (arising from reporting biases) for each synthesis assessed.	Not applicable
Certainty of evidence	22	Present assessments of certainty (or confidence) in the body of evidence for each outcome assessed.	13
**DISCUSSION**			
Discussion	23a	Provide a general interpretation of the results in the context of other evidence.	13–15
	23b	Discuss any limitations of the evidence included in the review.	15
	23c	Discuss any limitations of the review processes used.	15
	23d	Discuss implications of the results for practice, policy, and future research.	13–15
**OTHER INFORMATION**			
Registration and protocol	24a	Provide registration information for the review, including register name and registration number, or state that the review was not registered.	15
	24b	Indicate where the review protocol can be accessed, or state that a protocol was not prepared.	15
	24c	Describe and explain any amendments to information provided at registration or in the protocol.	Not applicable
Support	25	Describe sources of financial or non-financial support for the review, and the role of the funders or sponsors in the review.	17
Competing interests	26	Declare any competing interests of review authors.	17

Section and Topic	Item #	Checklist item	Location where item is reported
Availability of data, code and other materials	27	Report which of the following are publicly available and where they can be found: template data collection forms; data extracted from included studies; data used for all analyses; analytic code; any other materials used in the review.	Data extracted from studies

From: Page MJ, McKenzie JE, Bossuyt PM, Boutron I, Hoffmann TC, Mulrow CD, et al. The PRISMA 2020 statement: an updated guideline for reporting systematic reviews. BMJ 2021;372:n71. doi: 10.1136/bmj.n71

For more information, visit: http://www.prisma-statement.org/
